# Exposed endoscopic full-thickness resection with reopenable clip-over-the-line method for a duodenal neuroendocrine tumor

**DOI:** 10.1055/a-2222-8259

**Published:** 2024-01-09

**Authors:** Hitoshi Mori, Noriya Uedo, Satoki Shichijo, Tomoki Michida, Ryu Ishihara

**Affiliations:** 1 53312 Department of Gastrointestinal Oncology, Osaka International Cancer Institute, Osaka, Japan

The nonexposed endoscopic full-thickness resection (EFTR) device is available for endoscopic removal of duodenal subepithelial tumors, but the large application cap may make it difficult to resect lesions located in complex areas of the duodenum. Moreover, to insert the device into the duodenum, balloon dilation is often required, thus resection of a lesion behind the pyloric ring is difficult. Exposed EFTR can remove lesions in difficult sites; however, a secure closure method for the full-thickness wound is necessary for the success of the procedure. We report a case of exposed EFTR and full-thickness wound closure by reopenable clip-over-the-line method 1 for duodenal neuroendocrine tumor (NET).

An 80-year-old woman was diagnosed with a 7-mm subepithelial lesion just behind the pyloric ring ( Fig. 1 **a** ). Biopsy showed duodenal NET and she was referred to our hospital. At the time of endoscopic inspection before treatment, only a biopsy scar was identified by white-light endoscopy. However, mini-probe endoscopic ultrasound revealed a hypoechoic mass, measuring 3.9 × 1.8  mm, in the deep submucosa ( Fig. 1 **b** ). We decided to perform exposed EFTR, rather than endoscopic submucosal dissection, because there was no space between the lesion and the muscularis propria on endoscopic ultrasound. Moreover, with the nonexposed EFTR device, complete resection of the lesion would be difficult due to the limited working space of the duodenal bulb near the pyloric ring.

**Fig. 1:**
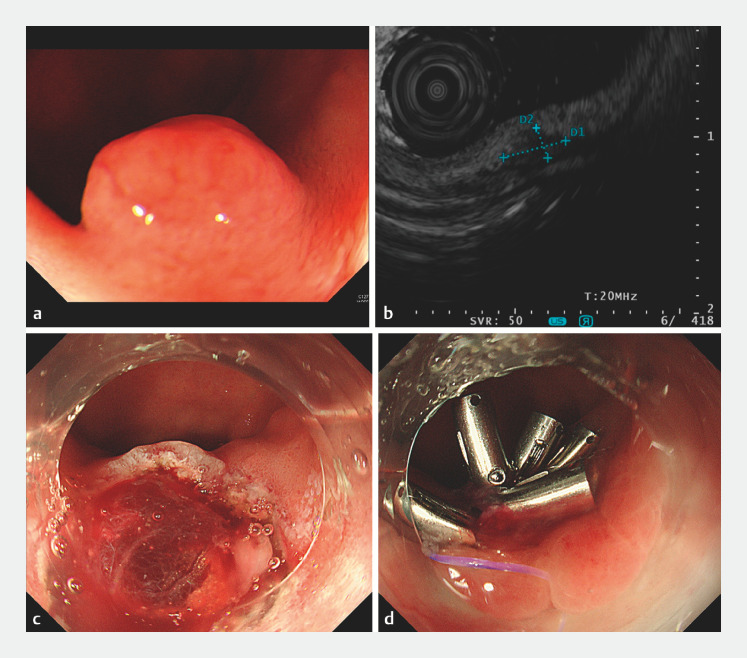
Exposed endoscopic full-thickness resection and full-thickness wound closure for a duodenal neuroendocrine tumor. **a** Endoscopic detection of the lesion at a previous hospital. **b** Mini-probe endoscopic ultrasound image showing a hypoechoic lesion in the deep submucosa. **c** The full-thickness resection wound. **d** Wound closure by the reopenable clip-over-the-line method.

Exposed EFTR was performed using a single-channel endoscope (GIF-H290T; Olympus, Tokyo, Japan) with transparent hood and a Flush knife BT 2.0 (Fujifilm, Tokyo, Japan) under general anesthesia. Clip-and-line traction (3–0 polyester suture line) was used to facilitate muscle incision and subtumoral dissection. After en bloc resection of the tumor, the muscle defect was closed by reopenable clip-over-the-line method using a 4–0 nylon suture line ( Fig. 1 **c,d** , Video 1 ). The total procedure time was 60 minutes.

Exposed endoscopic full-thickness resection with reopenable clip-over-the-line method for a duodenal neuroendocrine tumor.Video 1

The patient was discharged uneventfully. Histological examination confirmed a NET G1 with submucosal invasion (800 μm) ( Fig. 2 ). The vertical and horizontal margins were negative, with no lymphovascular involvement.

**Fig. 2:**
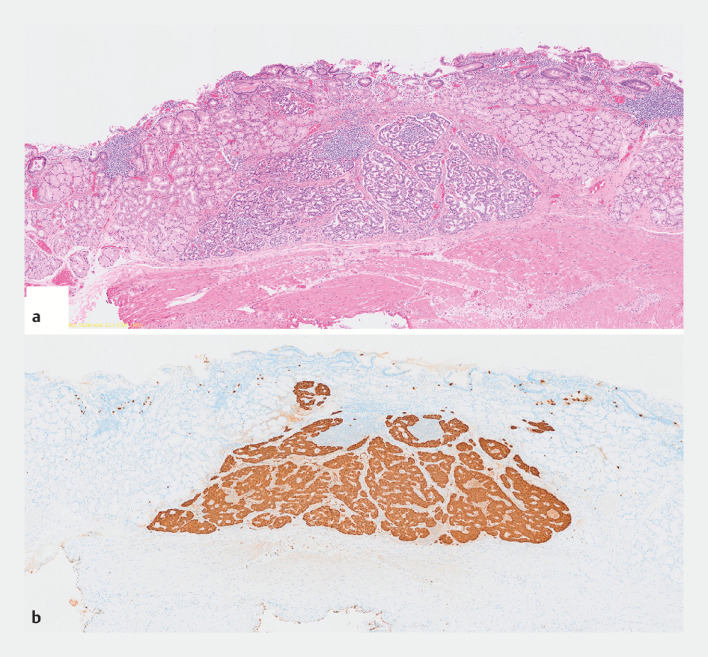
Histological findings of the lesion. **a** Hematoxylin and eosin staining. **b** Positive immunostaining with chromogranin A.

Endoscopy_UCTN_Code_TTT_1AO_2AC

## Notes

Endoscopy E-Videos https://eref.thieme.de/e-videos
*E-Videos* is an open access online section of the journal *Endoscopy* , reporting on interesting cases and new techniques in gastroenterological endoscopy. All papers include a high-quality video and are published with a Creative Commons CC-BY license. Endoscopy E-Videos qualify for HINARI discounts and waivers and eligibility is automatically checked during the submission process. We grant 100% waivers to articles whose corresponding authors are based in Group A countries and 50% waivers to those who are based in Group B countries as classified by Research4Life (see: https://www.research4life.org/access/eligibility/ ). This section has its own submission website at https://mc.manuscriptcentral.com/e-videos .
